# Lactation support in neonatal intensive care units in Germany from the mothers’ perspective – a mixed-method study of the current status and needs

**DOI:** 10.1186/s12884-024-06339-9

**Published:** 2024-04-16

**Authors:** Isabella Schwab, Ricarda Wullenkord, Friederike Eyssel, Till Dresbach, Nadine Scholten, on behalf of Neo-MILK, Andreas Müller, Martin Hellmich, Nicole Ernstmann, Antje Hammer, Angela Kribs, Juliane Köberlein-Neu, Katharina Lugani, Eva Mildenberger, Jens Ulrich Rüffer, Katja Matthias, Daniel Klotz, Anne Sunder-Plaßmann, Daniel Wiesen, Dirk Horenkamp-Sonntag, Iris Klein, Melanie Klein, Christoph Rupprecht, Laura Schleich, Anke Kurz

**Affiliations:** 1 Institute of Medical Sociology, Health Services Research, and Rehabilitation Science, Chair for Health Services Research University of Cologne, Faculty of Medicine and University Hospital Cologne, Eupener Straße 129, 50933 Cologne, Germany; 2 CITEC Center for Cognitive Interaction Technology, Bielefeld University, Inspiration 1, Bielefeld, 33619 Germany; 3 Department of Neonatology and Pediatric Intensive Care, Children’s Hospital, University of Bonn, Venusberg-Campus 1, 53127 Bonn, Germany

**Keywords:** VLBW infants, Preterm birth, MOM, Mother´s own milk, Lactation, NICU, Neonatal intensive care unit, Neo-MILK, Lactation support

## Abstract

**Background:**

Establishing successful lactation in mothers of very low birth weight (VLBW, <1500g) infants requires structured lactation support. Little is known about mothers’ perspectives on lactation support in German neonatal intensive care units (NICUs).

**Methods:**

This paper features a convergent mixed-method approach that includes a retrospective, cross-sectional questionnaire and interview data to showcase mothers’ perceptions of lactation support in NICUs. Content analysis of the interviews (*n* = 12) and a descriptive analysis of quantitative data (*n* = 533) were performed to illustrate the current status and need for lactation support in German NICUs.

**Results:**

The results show that lactation support in German NICUs is often inadequate and does not comply with recommendations based on the existing literature to encourage pumping and breastfeeding in mothers. The data imply that even if lactation is successfully initiated in most cases, it is often not maintained over time, which may be due to a lack of personal support and consistent information.

**Conclusion:**

The overall structures and institutional guidelines for lactation support should be encouraged to promote nutrition with mother´s own milk in German NICUs.

**Supplementary Information:**

The online version contains supplementary material available at 10.1186/s12884-024-06339-9.

## Background

In Germany, more than 10.000 children with very low birth weight (VLBW, < 1500g) are born every year [1]. The availability of mother’s own milk (MOM) in neonatal intensive care units (NICUs) is particularly important for VLBW infants due to its multiple positive effects on their health outcomes [2, 3]. These include a reduced risk of bronchopulmonary dysplasia (BPD), late onset sepsis, and necrotizing enterocolitis (NEC) [2, 4]. To achieve an effective initiation of lactation and to support mothers during the lactation process after giving birth to a VLBW infant, structured lactation support is required [5, 6].

The current literature provides recommendations on which interventions support establishing lactation in mothers of VLBW infants [7, 8]. To initiate lactation after preterm birth, mothers need to express milk as soon as possible, at best, within 6 h after birth [9]. Infants of mothers who initiate lactation later are at a higher risk of not being exclusively fed with MOM [10]. To facilitate this early lactation initiation, mothers need to be informed about the benefits of human milk, including MOM and donor human milk, ideally before birth [11]. In addition, preterm mothers should be instructed in manual milk expression as well as the use of an electric pump [12, 13]. Early instruction is particularly important to avoid formula feeding and ensure that colostrum is the first feed that preterm infants receive [14]. To foster continued feeding with MOM, a milk volume of at least 500ml/day should be reached by day 14 post-partum [15]. Moreover, the transition from pumping to breastfeeding showed to be crucial for achieving prolonged breastfeeding for the first six months of life and is recommended by the World Health Organization (WHO) [16, 17]. Early and prolonged skin-to-skin contact raises maternal oxytocin levels and has been shown to also be important for lactation [18, 19]. This includes family centered care, which allows the mother or parents to see and touch their child at all times [20].

In addition, taking psychological issues into account is particularly important when aiming to initiate and/or enhance lactation in preterm mothers, as premature birth can lead to psychological problems and/or aggravate pre-existing psychological conditions [21, 22]. A variety of psychological factors such as post-partum depression can influence breastfeeding and lactation behaviour [23, 24]; however, negative breastfeeding and/or lactation experiences can lead to and/or aggravate mothers’ psychological issues [25], especially if the experience does not meet the expectations [26]. It is, therefore, important to consider the mutual relationship between the mothers’ psychological well-being and lactation as well as breastfeeding. Institutional lactation support should aim to consider both the maternal psychological and physiological resources after preterm birth. To date, little is known about how mothers of VLBW infants experience lactation support in German NICUs.

This study aims to examine lactation experiences and perceived (emotional) challenges among mothers of VLBW infants in German NICUs. The data presented in this paper were collected within the Neo-MILK project, which intends to provide human milk for every VLBW infant from the first day of life in German NICUs [27]. This paper examines the key objectives of lactation support in the NICU in order to illustrate the status quo of lactation support in German NICUs from the mothers' perspective, as recommended by the literature. Quantitative and qualitative data will be combined in a mixed-method approach.

## Methods

Both data collections were performed as part of the Neo-MILK study. This study was funded by the Innovation Fund of the Joint Federal Committee (funding code: 01NVF19027) and registered in the German Register of Clinical Trials (ID: DRKS00024799, date of registration: 04/05/2021). It received a positive ethical vote from the University Hospital Cologne (20–1547) and Bielefeld University (2020–147). The interview questions and the quantitative questionnaire were developed in parallel and reviewed by both qualitative and quantitative research teams. Data collection occurred in quick succession and analyses were mostly performed in parallel. Therefore, this study applies a convergent mixed-method research design [28].

## Quantitative questionnaire

### Data collection

Quantitative data were obtained from a written, anonymous survey of mothers of preterm infants with a birth weight less than 1500g, 6–24 months after birth. The time point was chosen to minimize the probability of re-traumatization [29]. The self-developed questionnaire contained questions on maternal (e.g., age, education, previous experience with pumping, number of children) and neonatal (e.g., gestational age, birth weight) factors; birth setting (e.g., caesarean section, complications during birth), and lactation support (e.g., whether they were given information about MOM, pump instructions, or manual milk expression). Data were collected from June to August 2021 in cooperation with four statutory health insurance companies (AOK Rhineland/Hamburg, DAK, Pronova BKK, and TK).

### Data analysis

A total of 600 mothers participated in the survey, representing a response rate of 31,67%. After excluding data from mothers who failed to meet the inclusion criteria, the final sample included *n* = 533 cases. In order to describe the current status quo, nominal data is presented in percentages. In the case of Likert scales (six-point), means and standard deviations (SD) are presented. All statistical analyses were performed using STATA 16.

## Qualitative interviews

### Data collection

Qualitative interviews were conducted with* n* = 12 mothers of preterm infants with a birth weight of below 1500g. Recruitment methods included an Instagram post on the Neo-MILK Instagram account and information distribution about the ongoing study on the NICUs participating in the Neo-MILK projekt at the time of the data collection. Interested mothers then contacted the responsible researchers, were screened regarding the exclusion criteria (see below) and appointments were made if the requirements were met until the previously set goal of *n *= 12 participants was reached. Mothers were interviewed 3 to 12 months after their child had been discharged from the hospital to balance re-traumatization risk and the ability to remember sufficient details. One mother of whose twins only one survived and who wanted to participate in the interview was excluded in order to avoid re-traumatization. Due to the COVID-19 pandemic, interviews were conducted remotely via GoToMeetings and the audio was recorded. The interviews were held in the German language. To prevent potential data loss due to equipment failure, a transcript writer was present during the interviews, given the mothers had provided consent for this. The semi-structured interviews were conducted by a psychologist to alleviate the potential experience of psychological strain. The interview guide consisted of 113 questions, covering the topics of breastfeeding intention, current breastfeeding and pumping behavior, breastfeeding and pumping attitudes and related norms, infants’ stay at NICU (e.g., framework data, breastfeeding and pumping behavior during the stay), lactation and breastfeeding support (e.g., by hospital/NICU staff and partner), psychological stress factors, gender role orientation, previous breastfeeding and pumping behavior (i.e., for previous children), opinions and preferences regarding breastfeeding apps, and demographic questions (such as age, vocational status, and religion). A translated version of the interview guide including all questions that were asked can be found in the supplemental material.

### Data analysis

The audio files were transcribed and anonymized for follow-up qualitative content analysis [30] by a transcription office and were coded by two trained research assistants according to a previously developed coding scheme (Additional files 1 and 2). For all key objectives of lactation support, positive and negative anchor examples were identified, with positive anchor examples illustrating cases in which the lactation support was consistent with its respective objective, whereas negative anchor examples reflected cases in which the criteria were not met. More specifically, we focused on such quotations that gave additional insight into the reasons why the data presented a certain way, such as offering explanations. The quotations showcased in this paper were translated to English. Quotations were selected to illustrate a wide range of perceptions of mothers with different experiences in lactation support. If necessary, quotations were redacted for clarity, which is indicated by square brackets. The usage of caps lock in the quotations indicates special emphasis put on certain words.

## Results

Information on sociodemographic characteristics of both data is given in Table 1. Results are structured in accordance to the aforementioned key objectives to foster lactation (Table 2).

**Table 1:** Sociodemographic data

**Quantitative data**	
	*n*, (Mean; SD [min-max])
Maternal age	500 (34.1; 4.9 [19-54])
Gestational age (weeks)	519 (28.6; 3 [22-36])
	*n*, (%)
Educational Level	
Without a graduation	11 (2.1%)
Lower secondary school	52 (9.8%)
Secondary school	119 (22.3%)
Higher education entrance qualification	124 (23.3%)
University degree	215 (40.4%)
Missing	12 (2.3%)
Native language German	
Yes	413 (77.5%)
No	110 (20.6%)
Missing	10 (1.9%)
**Qualitative data**	
Maternal age	12 (34.5; 3.59) [29 – 40]
Children’s age at time of data collection (months)	Corrected (7.04; 2.56) [3-11]
	Uncorrected (9.54; 2.79) [5-14]
Educational Level	
Without a graduation	0 (0%)
Lower secondary school	0 (0%)
Secondary school	1 (8.33%)
Higher education entrance qualification	10 (83.33%)
University degree	1 (8.33%)
Missing	0 (0%)
Native language German	
Yes	12 (100%)
No	0 (0%)
Missing	0 (0%)

**Table 2:** Key objectives of lactation support

1. Informing all mothers and parents with a risk of premature birth about breastfeeding and pumping
2. Skin-to-skin contact in the delivery room with gradations (If skin-to-skin contact is not possible in the delivery room, touching the child and at least seeing the child directly after birth)
3. Initiation of lactation within one to four hours after birth, latest within six hours after birth with the gold standard to combine pumping and manual milk expression
4. Colostrum as the first feeding and no formula feeding
5. Maintain lactation with a pumping frequency of at least eight to ten times in 24 h three days after birth and reach at least a milk volume of 500 ml/day on day 14 days post-partum
6. Continuously checking maternal need for lactation support to enable early recognition of lactation problems and motivate mothers
7. Unlimited access to the child
8. Continuous, regular skin-to-skin contact
9. Early breast-to-mouth contact and transition to breastfeeding
10. Mother’s own milk as the gold standard

### 1. Informing all mothers and parents with a risk of premature birth about breastfeeding and pumping

Quantitative data show that more than one third of the mothers (37%) were not informed about the importance of MOM before birth. After birth, 22% received no information about MOM. Cross tables illustrate that 10% of the mothers (*n* = 53) did not receive any information on MOM either before or after birth.

In the interviews, mothers reported to have only rarely been informed of the importance of MOM before giving birth. Nevertheless, there were also examples of where information was more prioritized:

*"Before birth, I had a conversation with a neo-doctor, who then also addressed the issue or asked me […] how my attitude towards it would be. And […] I don't know whether on the first or second day up on the ward, the topic was addressed again by a doctor."* (M2)

Some mothers received rather brieft information about the topic after birth:

*"Not at all before birth. After birth, they said [it], but there was no big consultation about it."* (M3)

However, mothers also reported that they did not receive any information about the importance of MOM either before or after birth.

### 2. Skin-to-skin contact in the delivery room with gradations (if skin-to-skin contact is not possible in the delivery room, touching the child and at least seeing the child directly after birth)

More than half of the mothers reported having had the first skin-to-skin contact later than three hours after, but within the first day of birth (62%). While 28% of the mothers were able to see their child directly after birth, fewer mothers were able to have physical contact: 12% were able to touch their child directly after birth and only 5% had early skin-to-skin contact (Table 3).

**Table 3:** First contact between mother and child

	First time seeing the child after birth (n, %)	First time touching the child after birth (n, %)	First time of skin-to-skin contact after birth (n, %)
Directly after birth	149 (27.95%)	64 (12.01%)	29 (5.44%)
Within 3 h after birth	129 (24.20%)	229 (42.96%)	102 (19.14%)
Later than 3 h after birth	99 (18.57%)	145 (27.02%)	111 (20.83%)
Within the first day after birth	99 (18.57%)	81 (14.82%)	220 (41.28%)
Later	53 (9.94%)	14 (2.63%)	68 (12.75%)
Missing	4 (0.75%)	3 (0.56%)	3 (0.56%)

To deepen these insights, the interview data illustrate that some mothers were not able to have any contact with their child (including seeing their child) until the day after giving birth (within 24 h after giving birth):

*"About 13 h after that. […] What I found in retrospect very sad. […] I don't get it to this day why they did not show him to me for a second. I have a really big problem with a stranger [midwife] walking through the room with my child in her arms whereas I haven't seen my son for even a second. […]".* (M2)

One mother reported having encountered her child later than one day after birth. As a result, she reported being afraid to engage with her daughter:

*"Seeing her and touching her in the incubator was, I think, the next day when I was taken there in a wheelchair. But then I didn't dare to touch her yet, and I think two days after birth we were already allowed to cuddle her properly. But I didn't dare to do that either. I let my husband be the first. The first few days I wasn't quite myself yet."* (M8)

However, other respondents confirmed that they had the chance to encounter their child either directly after birth or within the same day:

*"I have seen him directly after the cesarean section, they lifted him up once. I was totally proud and thought: Wow. […]“* (M9)

### 3. Initiation of lactation within one to four hours after birth, latest within six hours after birth with the gold standard to combine pumping and manual milk expression

Only 36% of the mothers pumped within the first six hours of birth. Almost half of the mothers pumped later than 6 h after, but within 24 h after of (42%) (Table 4). Of those who initiated lactation, 57% combined pumping and manual milk expression. However, it should be mentioned that only 61% of the mothers received information about manual milk expression to win colostrum, of whom 87% then expressed colostrum and 13% did not.

**Table 4:** Timing of first pumping

Time of first pumping	n (%)
Within the first 2 h	30 (5.63%)
2–6 h	160 (30.02%)
6–24 h	222 (41.65%)
24–48 h	76 (14.26%)
3–7 days	24 (4.50%)
Later	2 (0.38%)
Not at all	15 (2.81%)
Missing	4 (0.74%)

The interviews deepen the findings about lactation initiation by demonstrating that the reason mothers did not express colostrum by hand was mostly because they did not receive any information about the importance of it and/or were not instructed to do so:

*"Nah, I just pumped. I didn't even know how to do the expression. I only learned that later during the lactation consultation."* (M9)

Apparently, communication issues between professionals from different wards emerged and posed a problem in that each had expected the other to instruct the mothers about manual milk expression:

*"[On the maternity ward] So this pump was put there, a pump set was supplied and [they said] "do it". I was completely overwhelmed with it. Because when I then arrived on the Neo [ward] two days later […] [they said] "Have you not brought any milk or colostrum or something? Don't you express?". And I was like: "WHAT? No, and there still is no milk at all.""* (M5)

In contrast, another mother reported being instructed in detail about colostrum and was reminded several times to either pump or express milk by hand:

*"They simply told us that this is enormously important, because there are still many, many, […] antibodies, […] and also all sorts of ingredients that a premature baby NEEDS, and that it is enormously important that you start pumping immediately after birth, because I really thought at that moment: Oh God, I have to recover after such a Caesarean section, and must somehow first get back on my feet, and my mind was not really on it at this moment, and it was good that there was always someone who came and said "How does it look?" and "Have you already pumped or expressed milk?""* (M4)

### 4. Colostrum as the first feeding and no formula feeding

More than half of the mothers reported formula feeding immediatley after birth (52%). One third of those mothers (33%, *n* = 80) stated that formula was provided as the main way to nourish their child during their stay in the NICU. In less than one-fifth (17%) of the cases, mothers indicated that their infants were provided with donor human milk while in the NICU.

The reported reason for why infants initially received formula was that the mothers had trouble producing (enough) milk:

*"In those first three or four days, when he didn't get any breast milk from me, it was some kind of premature neo-something milk and when I pumped and that wasn't enough for them, they mixed it first. I think it was on the seventh, eighth day that he got my milk only.“* (M3)

While some of the interviewed mothers were able to provide MOM for their children within the first week, others reported that they were already initially able to provide milk for their children:

*"I have to say it worked amazingly well. […] I was very proud and totally happy to be able to offer that to her then, despite the situation."* (M6)

### 5. Maintain lactation with a pumping frequency of at least eight to ten times in 24 h three days after birth and reach at least a milk volume of 500 ml/day on day 14 post-partum

Three days after birth, one fifth of the mothers (24%) pumped fewer than 6–8 times per day. Over 53% pumped 6 to 8 times a day, and only 18% pumped more than eight times within 24 h. More than half of the mothers (59%) had a milk volume of less than 500 ml/day by day 14 post-partum.

Although most mothers reported having initiated lactation within the first two hours after birth in the interviews, there was a contrasting experience in a mother who initiated pumping within the first 24 h of giving birth:

*“I think that was even maybe on the next day. […] Which I find strange now in retrospect, because you actually/ so many do it directly in the delivery room. But I think that it was on the next day, when I was able to sit up again.”* (M8)

Regarding the frequency of pumping in the first three days after giving birth, the mothers’ experiences differed between every two to three, every three to four, and every four to five hours. Interview data implied that pumping frequency varied in some cases due to inconsistent information given to the mothers, which they perceived as confusing and/or frustrating:

*"They made diverging statements. The nurse on the ward who gave me the pump said: "pump every four hours, that's enough." And at the intensive care ward, the nurse said: "In any case, every two to three and a half [hours] all day". These were just so very different statements and I was like: Huh? I don't know what to do."* (M3)

### 6. Continuously checking maternal need for lactation support to enable early recognition of lactation problems and motivate mothers

As lactation support can be seen as a multi-faceted approach of physiological and psychological support for lactating mothers, indicators of both dimensions are shown here.

In the quantitative data, more than one third of the mothers received no personal guidance on breastfeeding and/or pumping (39%). Most mothers who received personal guidance rated it as very good or good (73%). Breastfeeding or pumping problems were reported in 73% of the cases.

In the qualitative interviews, mothers were asked about personal guidance regarding breastfeeding and pumping. Some reported satisfactory personal guidance:

*"[…] I thought that was actually quite good. The nurse really, REALLY explained it very well. She was, I don't know if she was there until the end, but she was there for a relatively long time. She showed me the settings and how to scale it up and down and everything."* (M1)

Some mothers did not receive any personal guidance at all:

*"So for pumping there was no guidance. That did not take place."* (M2)

Some of the qualitative responses implied that personal guidance did take place in general, but mothers were often supported at a time point that was deemed too late:

*"I think I pumped far too infrequently […], every maybe three, four hours, like that. No one had told me anything concrete about it. I learned it afterwards […], always found out everything AFTERWARDS."* (M2)

**Fig. 1:**
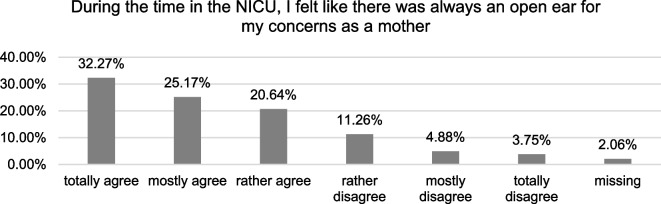
Distribution of agreement to the statement that they felt that there was always an open ear for the mothers´ concerns in the NICU

To illustrate the overall perception of care and support during the time in the NICU, mothers were asked if they agreed to the statement that they felt that there was always an open ear for their concerns as a mother in the quantitative questionnaire. Ranging from 1 (totally agree) to 6 (totally disagree), the mean agreement to this statement was 2.4, with an SD of 1.4. The distribution is shown in Fig. 1.

Indeed, the subjective feeling of the staff having an open ear significantly helped mothers cope with their current situation:

*"So actually [the] sympathetic ear itself [has helped the most]. [Even] when it's totally stressful […] and also when you're [at the hospital] for such a long time [it is important] that you can talk about problems. [The nursing staff] then said: 'Don't make yourself crazy."* (M4)

*"If I would talk to [the nursing staff] again, […] I would definitely give them feedback that I experienced the emotional support as really disproportionately good. They always had an open ear, they always built me up."* (M1)

However, some mothers reported divergent experiences with social and emotional support after birth. For instance, they missed being able to vent their feelings and to have a safe space for this. Others complained that institutional support, like psychological counseling, was not pointed out to them. The quote by M8 illustrates this:

*"No. I didn't know at the beginning that there was a psychologist in the hospital. I think that would have helped me at least for the first few days, just to somehow deal with the first shock.“*

### 7. Unlimited access to the child

The quantitative questionnaire contained no questions about the possibility of unlimited access to the child, but the mothers were asked about the options for rooming-in in their respective NICU. The possibility of rooming-in includes unlimited access to the child. Accordingly, this can be considered as an indicator of unlimited access. However, rooming-in was reported in only 3% of cases. Approximately half of the mothers (53%) reported staying at home during their child´s hospitalization.

To complement the quantitative perspective, we turned to findings from the semi-structured interview. In the interviews, we asked the mothers about whether they had the option of visiting the child without any restrictions. On a positive note, most mothers reported that they and their partner had unrestricted access to their child. Take, for instance, the quote from M2:

*"No, that was solved quite optimally. Me and my boyfriend, we were allowed to see our son 24 hours every minute and could also always call. So, we never felt like a nuisance or anything."*

On the other hand, mothers who reported restrictions on contact with their child explicitly linked these restrictions to COVID-19:

*“While the [children] were in NICU, there was a change in the hospital because the [covid] numbers went up, and then they said even though we have TWO [babies], only one person is allowed in a day. We weren't allowed to take turns either, only one was allowed in per day […]. We thought it was unfair because we had two children and got the same rights as for one child."* (M1)

Interview data further implied that the restrictions were perceived as a burden by the parents:

*"Quite awful. But I was told, it was COVID-related. We were allowed to cuddle one time a day. I think that was the worst part of the whole situation, yes."* (M3)

### 8. Continuous, regular skin-to-skin contact

The mothers were asked how they experienced the possibility of skin-to-skin contact in the NICU. Most were satisfied with the options for skin-to-skin contact (77%).

Regarding the opportunity to engage in regular skin-to-skin contact with the child, the experiences of the interviewed mothers were mixed, with some mothers reporting positive and some reporting negative experiences. Generally, skin-to-skin contact was supported by nurses and physicians in the NICU wards and hospitals, and the extent to which these institutions supported such contact often exceeded the mothers’ expectations:

*"So they lie there in their incubators and the nurses go there fully equipped with everything ad touch them very carefully and then we got her on the CHEST while kangarooing and were allowed to tube feed her and care for her, and change diapers, like a normal baby. So it was surprising for us that we were allowed to do that, even though she was so small."* (M1)

Similarly, mothers reported that they were allowed to have skin-to-skin contact in general. However, some admitted that they were reluctant to engage in skin-to-skin contact with the newborn, because they were afraid to eventually hurt the child.

*"The first time kangarooing was done by my husband. So we went there together and then the [nurse] said, "Do you want to cuddle?". And I was like, oh God, NO. That poor little being. They want to take him out of the incubator now and my husband said "YES" directly. In retrospect, I was so glad that he did that and I can't even UNDERSTAND nowadays that I didn't really want to do that. I was just afraid of him and of everything. […]".* (M9)

However, there were also reports of problems regarding the opportunity for skin-to-skin contact. One reason was that the NICU did not find time to arrange it at first:

*"At 10 o'clock was [the birth] and I think the first time I was brought over with the bed was in the late afternoon. But the ward didn't arrange it with the NICU, so they said: "No, it doesn't fit at all that you are there now". So I saw her I think once briefly and then they drove me away again. I think we had this game again the next day and then I said at some point "No matter what happens, I have to run over there myself now". Before it was physically not possible. On that day we kangarooed for the first time and spent more time together."* (M5)

Another reason were restricted visiting times due to COVID-19, a problem that was further aggravated if the mothers were already discharged and had to commute:

*"You then had two hours to kangaroo and if another mother sat there to kangaroo, you were often not allowed to go to the child, because it was too crowded due to the covid-19 regulations. The kangaroo time was then not made up for. I found it stressful, because if you were stuck in a traffic jam or something, you always had that time breathing down your neck: that's my kangarooing time going to waste. I was sometimes crying in the car. Or so happy to see my daughter and then drive there and the door is closed."* (M10)

### 9. Early breast-to-mouth contact and transition to breastfeeding

Quantitative data on early breast-to-mouth contact was not available. Nevertheless, the mothers were asked in the questionnaire whether they breastfed their child, which can be an indicator of whether breast-to-mouth contact was achieved in any form. This transition from pumping to breastfeeding was achieved in almost half of the mothers (46%).

Interview data implied that mothers were rarely supported in the transition from pumping to breastfeeding, and that early breast-to-mouth contact was also rarely supported:

*"[…] In the NICU when I asked if I could breastfeed [they told me]: "No, you can't, yet", and I still don't understand why. He didn't have to drink at all. He could have just sniffed or sucked and I think I just did that at some point under my snuggle blanket. I will never forget my friend saying „Today you go there and you just DO it. It ‘s YOUR child, you're allowed to do that", and that was incredibly good, and through that he felt me somehow. To this day, I don't understand why they weren't open to support this concrete closeness more."* (M3)

Similarly, when the complete transition from pumping to breastfeeding was realized during the hospital stay, it was apparently due to the mother actively asking for support and insisting on breastfeeding:

*"There are [fixed] feeding times, so there is no chance for need-oriented feeding, […]. I always said: I have to have him with me and I'm sure I'll get him to breastfeeding, and I fought and fought and fought, and actually […] because I was such a huge pain in the ass to them [the hospital staff], they took me in five days before [his] discharge and I could completely breastfeed him 24 h later.“* (M3)

Still, some mothers did not complete the transition from pumping to breastfeeding and received no support in trying to do so which in some cases led to a quick decline in the amount of breast milk they were able to provide after they were discharged from the hospital themselves:

*"Quite quickly, when we were at home. […] Somehow it just became less and less. […] I have tried everything. But I think perhaps it is still a bit different in such a situation with such an extremely premature birth, and the clinic was 70 km away from our place, and with the commute, that is of course again stress and takes a lot of time, […] what I really would have needed was proper help or advice."* (M8)

### 10. Mothers own milk as the gold standard

**Fig. 2:**
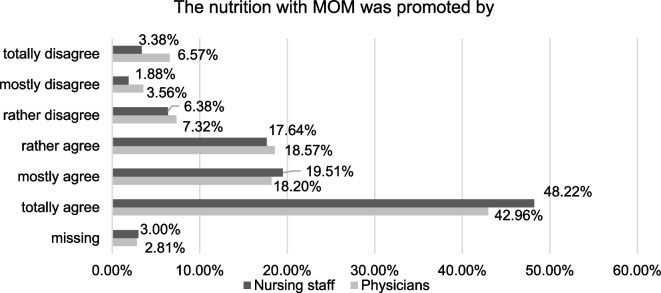
Distribution of the agreement to the statement that nutrition with MOM was promoted by the NICU staff

In the survey, mothers were asked whether they agree with the statement that nutrition with MOM was promoted by physicians and nursing staff. Ranging from 1 (totally agree) to 6 (totally disagree), mean agreement was 2.3 (SD = 1.5). The detailed distributions of both the variables are shown in Fig. 2. However, 30% of mothers reported that an exclusive nutrition with MOM was achieved.

The interview data illustrated that many mothers were already highly intrinsically motivated to provide MOM for their children from the beginning:

*"In general, it was ALWAYS clear to me when I would have a child that I wanted to breastfeed. So for me nothing else was worth consideration."* (M2)

In in most cases, the hospitals further supported this notion by explicitly communicating MOM as the gold standard for infant nutrition:

*"It was always clear to me that I wanted to try, and I was very, very determined when she was born that breast milk was the best thing for her. This was also mentioned again and again on the premature infant ward that breast milk is the best, especially for premature infants, and therefore it was important to me to achieve it in any case, at least as far as possible."* (M10)

In some cases, they explicitly advocated against formula as well:

*"[…] In the hospital, they told me that [breast milk] was easier to digest than bottle feeding or formula. I really didn't think about that before."* (M7)

In a few cases, mothers reported that the hospital did not give any information about the fact that MOM would have been the preferable form of nutrition:

*"I knew about the importance of breast milk. But I think […] if it hadn't been my third child, it wouldn't have been clear to me […]."* (M3)

## Discussion

This study utilized a mixed-method approach, including qualitative and quantitative data, to examine mothers’ perceptions of the lactation support they experienced around the birth of their VLBW infants. The survey and interview data were analyzed and structured according to the key objectives recommended for lactation support in NICUs. Overall, our data show the need for improvement in lactation and breastfeeding support in German NICUs and provide insight into the specific perceptions of mothers.

While previous studies have shown the importance of providing information on the relevance of human milk to the mother prior to the birth of a preterm infant [11, 31], in many cases, mothers did not receive any information either before or after delivery. Early lactation initiation is one of the main factors leading to successful lactation after preterm birth [11]. Our data indicate that more than half of the mothers did not initiate pumping until 6 h after birth. Delayed first pumping can lead to insufficient milk supply; thus, timing is a central factor in lactation support in the NICU [32]. Interview data indicate that delayed pumping was often due to mothers not being clearly instructed regarding the usage of the electric pump, despite its importance in achieving efficient use [33]. To enable colostrum to be the first feed for infants, manual milk expression should be combined with pumping after birth [14, 34]. Quantitative data show that many mothers were not aware of manual milk expression and its methods. However, when mothers received information, nearly all of them applied it, emphasizing the need for guidance. In our study, less than half of the mothers reached the required milk supply of more than 500 ml/day on day 14 post-partum [15]. This may explain the frequent formula-feedings reported in our sample. Low milk supplies could be due to late initiation of pumping, but also low pumping frequency within the first days, which was sometimes fewer than six and often fewer than eight times in a 24-h period, despite the requirement for frequent milk expression to maintain milk supply [9]. The qualitative data indicate that this is based on misinformation, inconsistent information, or delayed information from hospital staff. This may further explain the high rate of mothers who reported problems with pumping or breastfeeding in our data.

Although the importance of skin-to-skin contact or, if not possible, touching or seeing the newborn is well known for improving lactation success [19, 35, 36], only 5% of the mothers reported direct skin-to-skin contact after birth, 12% touching, and 28% eye contact. The qualitative data suggest that mothers could suffer from having no chance to have skin-to-skin contact with their child directly or soon after birth, indicating its importance for maternal well-being. However, the majority of mothers expressed satisfaction with the possibility of skin-to-skin contact during their child's stay in the hospital.

Furthermore, it is crucial for mothers to receive emotional and (professional) psychological support after preterm birth to protect their mental health [37]. Most mothers had a positive perception of the emotional support provided by the NICU staff; however, the interview data suggest that there may be room for improvement, as many mothers were not aware of the availability of professional psychological counselling. Given that stress can negatively affect lactation, the need for improvement is further emphasized [38].

Almost half of the mothers in the quantitative sample achieved the transition from pumping to breastfeeding, which has been shown to lead to a longer breastfeeding duration [16]. There is uncertainty as to whether the mothers in our quantitative sample chose to initiate breastfeeding rather than utilizing pumping or whether they received adequate support to facilitate the transition. However, the interview data suggest that a lack of support can hinder the transition, indicating the need for improvements to enable long-term feeding with MOM.

In summary, lactation support in NICUs in Germany shows room for improvement. On the one hand, feeding with MOM is proposed as the gold standard; on the other hand, our results reflect a lack of support for lactation and breastfeeding in actual clinical practice. Considering that beds frequently have to be blocked due to staff shortages [39], the indicated lack of lactation support in our data could be at least partly attributed to such structural issues. One central issue that repeatedly arose in our results was the lack of (personal) guidance, especially concerning consistent information through all hospital wards. This indicates missing structures and policies regarding lactation support across multidisciplinary teams of obstetrics, gynecology, and neonatology, which have been shown to be particularly relevant [40]. Previous research has shown that a consistent information policy across the various stakeholders as well as an environment that supports nutrition with human milk are crucial for lactation support and breastfeeding promotion [41–43]. These may therefore be factors to focus on when designing future interventions for lactation support in German NICUs.

### Limitations

Even though our study provided relevant insights into the status quo of lactation support in German NICUs and offers possible solutions for some of the existing issues, some limitations must be considered: Firstly, our study predominately provides data on mothers who initiated pumping. Only 3% of the mothers in the quantitative sample and none included in the qualitative sample did not initiate pumping at all. However, as mothers of preterm infants are more likely to initiate lactation than term mothers, the high initiation rate in our data hardly deviates from this [44]. Future research should, therefore, specifically target mothers of VLBW infants who do not initiate lactation to consider their insights into the topic and their reasons for not initiating lactation.

Secondly, since there are no validated and established scales on stress and/or psychological strain due to pumping and breastfeeding, especially for preterm infant mothers, self-generated items were used in the quantitative questionnaire. An expert team developed and revised these items. However, the specific choice of items may still have influenced the results, and future research should focus on validating scales for these topics. Thirdly, the interviews had to be conducted online due to COVID-19, which may have led to slightly varying answers compared to in-person interviews because the situation may feel less personal than a face-to-face interview. In addition, the interview contained many questions that may have felt more tiring in an online setting, which might have led to shorter answers at the end of the interview compared to the beginning.

Furthermore, certain particularly vulnerable populations (e.g., in this case, mothers of twins of which one child did not survive) were not contacted for the study and are not represented in the samples. Future studies could target more vulnerable populations to gain valuable insights into the topic. This also allies to mothers with low educational status or migration background, wo were hardly represented in our data.

Finally, the mothers were contacted six months after giving birth. This might have led to recall bias due to mothers not recalling information correctly and/or over- and under-emphasizing certain aspects. However, as mothers who experienced premature birth are at a higher risk of depression, anxiety, and post-traumatic stress [37], this inclusion criterion was implemented in order to minimize possible re-traumatization after preterm birth.

## Conclusion

This study examined the lactation support at German NICUs from the mother’s perspective using a mixed-method approach, including a quantitative questionnaire and qualitative interviews with mothers of VLBW infants.

This study adds to knowledge about mothers´ perceived lactation support, showing broad areas for improvement. To enhance nutrition with MOM in VLBW infants, thereby preventing health and development risks, the key objectives of lactation support should be improved. Our findings indicate the need to introduce institutional guidelines and overall structures in lactation support across all involved hospital wards to support successful lactation in preterm mothers.

### Supplementary Information

**Supplementary Material 1.**

**Supplementary Material 2.**

## Data Availability

The datasets used and/or analyzed during the current study are available from the corresponding authors on reasonable request.

## Abbreviations

VLBW: Very low birth weight
MOM: Mother’s own milk
NICU: Neonatal intensive care unit
BOP: Bronchopulmonary dysplasia
NEC: Necrotizing enterocolitis
WHO: World Health Organization
