# Supplementary material for: Lactation support in neonatal intensive care units in Germany from the mothers’ perspective – a mixed-method study of the current status and needs
Source: BMC Pregnancy Childbirth. 2024 Apr 16;24:282. doi: 10.1186/s12884-024-06339-9 (PMC11022450; doi:10.1186/s12884-024-06339-9)
Supplement: Supplementary file 1 — Supplementary Material 1. [file 12884_2024_6339_MOESM1_ESM.pdf]

Additional file 1

Interview questions (English translation)

**Breastfeeding intentions and current breastfeeding/pumping behavior**
Open introductory question: How is it going at the moment, are you breastfeeding, perhaps pumping or even feeding your child with donor milk? How is breastfeeding going?
Did you already intend to breastfeed your baby during pregnancy? Why or why not?
When did you start looking into breastfeeding/pumping?
From whom did you first receive information about breastfeeding / pumping, and when? Or did you find out about it yourself?
If breastfeeding intentions exist: At what point did you decide that you wanted to try feeding your baby breast milk?
What would you describe as your main motivation to feed your baby breast milk? / What motivated you most to feed your baby breast milk?
If you stopped breastfeeding or pumping, when did you stop and why?
How long in total did you breastfeed and / or pump?
If not breastfeeding/pumping at all or stopped very early: What were the reasons why you did not breastfeed/pump or stopped very early?
If not breastfed/pumped at all or stopped very early: would you have actually wanted to continue, and what would you have needed to do so? (e.g., help, information, etc.).
What emotional significance does/did pumping have for you and your relationship with your child?
What emotional significance does/did breastfeeding have for you and your relationship with your child?
If you have weaned or stopped pumping: How did your environment (e.g., partner, friends, relatives) react?
**Breastfeeding attitudes and norms**
Open introductory question: What do you think about breastfeeding?
Were you breastfed?<br>Do you know if your mother was breastfed?<br>If you are in a relationship, do you know if your partner was breastfed?
Is breastfeeding or supplying breastmilk for your baby important to you personally? Why or why not?
What do you generally think about breastfeeding / pumping and the supply of breast milk to newborns / infants?
What do you think about providing newborns / infants with donor milk?
Now this is an example of a series of questions that may seem repetitive to you. Feel free to answer briefly or refer to a previous answer if there is overlap there. But first, let's get started.<br>What does your partner think about breastfeeding and pumping in general?
If you breastfeed/pump, what does your partner think about you breastfeeding/pumping?
What does your mother-in-law think about breastfeeding and pumping in general?<br>If you breastfeed/pump, what does your mother-in-law think about you breastfeeding/pumping?
What does your mother think about breastfeeding and pumping in general?
If you are breastfeeding/pumping, what does your mother think about you breastfeeding/pumping?
What do your other relatives think about breastfeeding and pumping in general?
If you breastfeed/pump, what do your other relatives think about you breastfeeding/pumping?
Do you know other women who breastfeed/pump?

What experiences have these women reported? Did it influenced you in your decision to breastfeed / not to breastfeed?
What do your friends or other mothers you know think about breastfeeding and pumping in general?
**Stay in the NICU: general data, breastfeeding/pumping behavior**
Open introduction: Your baby/ babies were born prematurely, so I have a few questions about that.
Were you in the NICU with your baby after he/she was born, and if so, for how long?
How long in total were you in the hospital with your baby?
When did you first see your baby after birth?
While your baby was in the NICU, were you allowed to see your baby at any time or were your visitation rights restricted? If so, in what way? How did you perceive these restrictions (e.g., were they burdensome or relieving)?
Did your baby receive woman's milk (i.e., breast milk or donor milk) from birth?
If yes: Did you provide your baby with breast milk yourself, or did your baby receive donor milk?
Did you express breast milk after birth? If yes: how (express, pump)? Approximately how often and when did you switch to other methods of milk expression, if applicable?
How did you feel during your first attempts to express breast milk?
Have you been educated about the special importance of colostrum for preterm infants? If yes: by whom?
Did you pump in the NICU? If yes: How soon after birth did you first pump? If no, why did you not pump?
If yes: Approximately how often did you pump during the first three days? Were there fixed time intervals?
If yes: Did you pump both breasts at the same time or one after the other?
Were you reminded to pump by doctors, nurses, or other hospital staff?
Were you allowed to see your baby while you pumped?
If your baby did not get breast milk from birth, did he or she get your breast milk or donor milk at a later time? If yes, from when on?
If donor milk: when were you first able to feed donor milk?
If breast milk (also after donor milk): When were you first able to feed your own breast milk? How was this done (e.g., by tube, by breastfeeding, or by pumping and feeding)? Do you feel like you would have been able to do so earlier?
Did the hospital give you the opportunity to go to your baby at night and feed him or her your breast milk or donor milk? If not, would you have liked to have such an opportunity?
Did you feel you were allowed to make your own decisions regarding breastmilk supply/donor milk supply for your baby and also to say you did not want things you were advised to do (e.g., feed donor milk, use nursing caps, etc.)?
Do you feel or have you felt pressured or pushed to breastfeed and pump against your wishes? If so, by whom?
It can happen that some children in the NICU are not yet in good health at the beginning. Was this the case with your child? If so, what were the problems?
**External breastfeeding and pumping support:**
Open introductory question: did anyone help you breastfeed or pump?
Did your partner assist you with breastfeeding, milk expressing, or pumping?
Have you been counseled about the importance of breast milk for preterm infant health? This includes counseling before birth, but also after.
Were you assisted in breastfeeding or expressing breast milk in the hospital, for example by explanations about milk pumps, latching on, etc.? If yes: By whom?
Were you satisfied with breastfeeding and pumping support in the hospital?
What tips or what support for breastfeeding and pumping from the doctors helped you the most?

What tips or what support for breastfeeding and pumping from nurses (in neonatology or gynecology wards) and midwives helped you most?
Is there anything you would have wished for in terms of advice or support to promote breastfeeding and pumping or breast milk production?<br>Did you feel comfortable with the doctors' guidance of the breastfeeding process/ pumping?
Did you feel comfortable with the nurses' assistance during the breastfeeding process / pumping?
Do or did you have negative feelings about breastfeeding or pumping? For example, some mothers feel shame, dejection, anger, guilt, or feel like they are failing. Have you experienced such feelings as well? If so, what have you done to change this and what has helped you?
Did you feel that you or your body was being instrumentalized in terms of breastfeeding and supplying breast milk? If so, did you feel this was negative or perhaps even necessary in the situation?
Did you feel that you or your body were being reduced to breast milk production?
Did you have any breastfeeding counseling after your hospital stay? If so, by whom? How confident did you feel about breastfeeding/pumping afterward?
What breastfeeding and pumping tips would you pass on to other mothers?
What tips would you have for medical and nursing staff to better assist mothers with breastfeeding / pumping?
Did you feel valued in your breastfeeding and pumping efforts? If yes: By whom?
If yes: Was this appreciation important to you? If applicable, especially in the early days? Why (e.g., motivation)?
Did you feel confused by conflicting information about breastfeeding and pumping from different sources? If yes: Do you have examples of conflicting information?
How do you feel while breastfeeding or pumping?
What do you think would have to be done to convince mothers to breastfeed especially premature babies or to pump breast milk for them?
What do you think would have to be done to convince mothers to accept donor milk for their babies?
What form of support or advice from doctors and nurses do you think is most important / for breastfeeding or providing breast milk for premature babies?
How easy did you find it to continue pumping/breastfeeding after discharge? Did you face any obstacles in doing so?
What support did you find or would you find helpful to continue breastfeeding / pumping at home after your stay in the hospital?
What information do you think is important when it comes to breastfeeding/breastmilk supply in general and especially breastfeeding/breastmilk supply for premature babies?
How did you get information about breast milk and breastfeeding, what were your main sources of information?
Did you network and exchange information with other breastfeeding mothers? If yes: online or in real life?
Did these exchanges help you? If yes: for what exactly and in which ways?
Were there any situations in which you felt particularly proud or especially strong as a mother after your baby was born prematurely? If yes: What were they?
**Psychological stress factors**
Did you feel psychologically burdened by the premature birth and everything that went along with it? If yes: By what in particular?
Do you currently feel particularly psychologically stressed?
If yes to one of the two questions: Did your psychological stress situation prevent you from providing your baby with breast milk? If yes: Through whom did you get help or are you getting help?

If you would like to share: Did you get diagnosed with a mental illness? If yes: when and which one?
Has breastfeeding or pumping helped you cope with your psychological distress situation?
**Gender role orientation**
Do you see breastfeeding as a natural duty of a mother?
Does your partner see breastfeeding as a mother's duty?
Does your partner help with household chores?
Does your partner help with the care of your baby/children in everyday life?
**Past breastfeeding and pumping behavior**
Do you have other children? If so, how old are they?
Were your other children also born prematurely?
Did you breastfeed your other children or pumped breast milk for them?
If you would have had breast milk to spare, would you have provided it as a donation for other babies, or did you actually donate breast milk?
**Breastfeeding apps**<br>Open introductory question: are you familiar with breastfeeding support apps?
If known: Do you use a breastfeeding support app yourself? If yes: Which one?
If no: Would you use a breastfeeding support app? How likely is it (in percent) that you would use a breastfeeding support app?
What features would you want in such an app?
How often or at what intervals should such an app remind you to breastfeed?
What information about breastfeeding and pumping should definitely be included in such an app?
**Sociodemographic questions**
How old are you?
Were you born in Germany? If no: In which country were you born?
If no: How long have you lived in Germany?
Are you in a relationship? If yes: With the father of the child or with another person?
If married/partnered, how many years have you been married/partnered?
If no: Are you a single parent or which parenting model between partners do you use?
Do you belong to a religious group? If yes, which one?
What level of education do you have?
Are you employed
If yes: What is your current employment status? (e.g. full-time / part-time / mini-job)
What was your last / is your current occupation?
If employed: Have you already returned to your job? If yes: How old was your child when you started working again?
If back at work: Why did you start working again? (e.g., financial reasons, enjoyment of work, securing position, etc.?)
If still breastfeeding/pumping and working: Does your employer support breastfeeding/pumping and provide you with the necessary breaks? *(Note. This relates to German working law in which workers are entitled to breastfeeding/pumping breaks)*
If still breastfeeding/pumping and working: How do you handle breast milk supply while you are at work?
At what point in your pregnancy did your baby come into the world? Please give your answer in the following format, e.g. 37 + 5. If you do not know the exact gestational age, give only the completed week of pregnancy: 31 here means 31 completed weeks, so birth at 32 weeks.
When did you know that your baby would be born prematurely?
How old is your baby now? If known, feel free to list both the uncorrected age and the corrected age.

**<u>Other</u>**
Is there anything else you would like to say / tell us that we did not ask for?
Finally, do you have any suggestions for us? What else should we ask about in further interviews with mothers?
