# Supplementary material for: Lactation support in neonatal intensive care units in Germany from the mothers’ perspective – a mixed-method study of the current status and needs
Source: BMC Pregnancy Childbirth. 2024 Apr 16;24:282. doi: 10.1186/s12884-024-06339-9 (PMC11022450; doi:10.1186/s12884-024-06339-9)
Supplement: Supplementary file 2 — Supplementary Material 2. [file 12884_2024_6339_MOESM2_ESM.pdf]

Additional file 2

Coding scheme (English translation)

Note. Some questions were shortened to put into the coding scheme, so the questions are not necessarily identical to the formulations in the interview guide, as the notes in the scheme were aimed to provide orientation for the coders to find the corresponding question in the interview guide.

Coding scheme maternal interviews Neo-MILK

Notes:

- If a woman does not give a specific answer to a topic and / or the question was not asked code with -99.
- Questions were not always asked in the order they are here or in the interview guide
- In some cases, answers to several questions were given in the context of a single question, then enter the answers accordingly for the appropriate questions. In this case, the respective questions were often skipped in the further course and / or the indication was given that the answer had already been given further in advance
- If you are unsure about a coding, write a comment on it and describe where the uncertainty comes from or what is unclear.

**Breastfeeding intentions and current breastfeeding/pumping behavior**	**Coding**
Open introductory question: How is it going at the moment, are you breastfeeding, perhaps pumping or even feeding your child with donor milk? How is breastfeeding going?	Rate categorially<br>Breastfeeding = 1<br>Pumping = 2<br>Bottlefeeding = 3<br>Complementary food = 4<br>Donor milk = 5<br>Complementary food & Breastfeeding = 6<br>Complementary food & Pumping = 7<br>Complementary food & Bottlefeeding = 8
Did you already intend to breastfeed your baby during pregnancy?	Rate categorial<br>yes = 1, no = 2
Why or why not?	Rate categorially<br>Because it is the best nutrition for my child = 1<br>Because it is the most natural nutrition = 2<br>Because it is the mothers' duty / simply part of it = 3<br>I have not made any thoughts about it = 4<br>No, because I fastly wanted to get back to my occupation = 5<br>Motivation through / orientation by the environment = 6
When did you start looking into breastfeeding/pumping?	Rate categorially<br>prior to pregnancy = 1<br>during pregnancy = 2<br>subsequent birth = 3<br>not at all = 4
From whom did you first receive information about breastfeeding / pumping, and when? Or did you find out about it yourself?	Rate categorially<br>Lactation consultant = 1<br>Midwife = 2<br>Nursing staff = 3<br>Physicians = 4<br>Family Members = 5

	Friends = 6<br>Other mothers at the station = 7<br>Self = 8<br>Gynaecologist (outside the hospital) = 10
If breastfeeding intentions exist: At what point did you decide that you wanted to try feeding your baby breast milk?	Rate categorially<br>prior to pregnancy = 1<br>during pregnancy = 2<br>subsequent birth = 3
What would you describe as your main motivation to feed your baby breast milk?	Rate categorially<br>general health of the child = 1<br>good for the immune system = 2<br>Bonding = 3<br>better development = 4<br>natural nutrition = 5<br>natural duty of the woman / simply part of it = 6<br>practical/cheap = 7<br>premature birth = 8
If you stopped breastfeeding or pumping, when did you stop?	Rate categorially<br>prior to discharge of the child from the hospital = 1<br>during the first month subsequent to discharge of the child from the hospital = 2<br>during the first two months subsequent to discharge of the child from the hospital = 3<br>during the first three months subsequent to discharge of the child from the hospital = 4<br>during the first four months subsequent to discharge of the child from the hospital = 5<br>during the first five months subsequent to discharge of the child from the hospital = 6<br>Six or more months subsequent to discharge of the child from the hospital = 7<br><br>Woman still breastfeeds / pumps = -99
If you stopped breastfeeding or pumping, why did you stop?	Rate categorically:<br><br>too little milk = 1<br>psychological strain = 2<br>too cumbersome / time-consuming = 3<br>health reasons of the mother = 4<br>health reasons of the child = 5<br>did not feel like it anymore = 6<br><br>woman did not stop = -99<br><br>If more than one factor is mentioned, code the deciding factor
How long in total did you breastfeed and / or pump?	Rate categorically<br>up to 2 weeks = 1<br>up to 4 weeks = 2<br>up to 6 weeks = 3<br>up to 2 months = 4<br>up to 4 months = 5

	up to 6 months = 6<br>more than 6 months = 7<br>Still breastfeeding = 8<br><br>not at all = -99
If not breastfeeding/pumping at all or stopped very early: what were the reasons why you did not breastfeed/pump or stopped very early?	Psychological reasons = 1<br>Too little milk = 2<br>too time consuming / too inconvenient = 3<br>Health reasons = 4<br>Did not intend to breastfeed / pump = 5<br>External advice to switch to powdered milk = 6
If not breastfed/pumped at all or stopped very early: would you have actually wanted to continue, and what would you have needed to do so? (e.g., help, information, etc.).	Psychological counseling = 1<br>preparatory support by the hospital for time after discharge = 2<br>social-medical aftercare = 3 Breastfeeding and lactation counseling = 4<br>More support from family / partner = 5<br>Access to necessary infrastructure (e.g. breast pump) = 6<br>Other factors = 7<br><br>If more than one factor is mentioned, code the most important factor.
What emotional significance(s) did pumping have for you and your relationship with your child?<br><br>code two variables, one for positive emotions and one for negative emotions	Categorial<br>Positive emotions:<br>emotional attachment/ closeness = 1<br>Satisfaction through certainty of the best possible developmental support = 2<br>relaxation = 3<br>Joy = 4<br>Appreciation of time spent together = 5<br><br>Negative emotions:<br>exhausting / stressful = 1<br>frustrating = 2<br>anxiety inducing = 3<br>induces sadness = 4<br><br>If more than one factor is mentioned, code decisive factor
What emotional significance(s) did breastfeeding have for you and your relationship with your child?<br><br>code two variables, one for positive emotions and one for negative emotions	Categorial<br>Positive emotions:<br>emotional attachment/ closeness = 1<br>Satisfaction through certainty of the best possible developmental support = 2<br>relaxation = 3<br>Joy = 4<br>Appreciation of time spent together = 5<br><br>Negative emotions:<br>exhausting / stressful = 1<br>frustrating = 2<br>anxiety inducing = 3<br>inducing grief = 4<br><br>see above.

If you have weaned or stopped pumping: How did your environment (e.g. partner, friends, relatives) react?	Negative = 1<br>Positive = 2<br>Neutral = 3
**Breastfeeding attitudes and norms**	
Open introductory question: What do you think about breastfeeding?	
Were you breastfed?	Categorial<br>yes = 1<br>no = 2<br>do not know = 3
Do you know if your mother was breastfed?	Categorial<br>yes = 1<br>no = 2<br>do not know = 3
If you are in a relationship, do you know if your partner breastfed?	Categorial<br>yes = 1<br>no = 2<br>do not know = 3
Is breastfeeding or breastmilk supply for your baby important to you personally? Why or why not?	Likert scale 5 levels (not at all important = 1, not important = 2, neither important nor unimportant = 3, important = 4, very important = 5).<br><br>Why: (categorical)<br>general health of the child = 1<br>good for the immune system = 2<br>bonding = 3<br>better development = 4<br>natural nutrition = 5<br>natural duty of the woman / simply belongs to it = 6<br>practical/beneficial = 7<br>premature birth = 8
What do you think in general about breastfeeding / pumping and the supply of breast milk to newborns / infants?	Categorial:<br><br>best nutrition for the child = 1<br>Important for the immune system = 2<br>important for development = 3 important for general health = 4 important for bonding = 5<br>Is part of it when having a child = 6
What do you think about providing newborns / infants with donor milk?	Categorical:<br><br>positive opinion about SM without further statement = 1<br>positive opinion about SM, but would not use it myself = 2<br>positive opinion about SM and would use it = 3<br>positive opinion about SM and has donated / would like to donate = 4<br><br>Unsure / need more information = 5<br>neutral attitude = 6<br>negative attitude = 7
What does your partner think about breastfeeding and pumping in general?	Negatively set = 1<br>Neutral set = 2<br>Positively set = 3

	Unknown = 4
If you breastfeed/pump, what does your partner think about you breastfeeding/pumping?	Negatively set = 1<br>Neutral set = 2<br>Positively set = 3<br>Unknown = 4
What does your mother-in-law think about breastfeeding and pumping in general?	Negatively set = 1<br>Neutral set = 2<br>Positively set = 3<br>Unknown = 4
If you breastfeed/pump, what does your mother-in-law think about you breastfeeding/pumping?	Negatively set = 1<br>Neutral set = 2<br>Positively set = 3<br>Unknown = 4
What does your mother think about breastfeeding and pumping in general?	Negatively set = 1<br>Neutral set = 2<br>Positively set = 3<br>Unknown = 4
If you are breastfeeding/pumping, what does your mother think about you breastfeeding/pumping?	Negatively set = 1<br>Neutral set = 2<br>Positively set = 3<br>Unknown = 4
What do your other relatives think about breastfeeding and pumping in general?	Negatively set = 1<br>Neutral set = 2<br>Positively set = 3<br>Unknown = 4
If you breastfeed/pump, what do your other relatives think about you breastfeeding/pumping?	Negatively set = 1<br>Neutral set = 2<br>Positively set = 3<br>Unknown = 4
Do you know other women who breastfeed/pump?	yes = 1<br>no = 2
What experiences have these women reported?	Negative = 1<br>Neutral / mixed = 2<br>Positive = 3
Has it influenced you in your decision to breastfeed / not to breastfeed?	Yes, negatively influenced = 1<br>Yes, positively influenced = 2<br>Not influenced = 3
What do your friends or other mothers you know think about breastfeeding and pumping in general?	Negatively set = 1<br>Neutral set = 2<br>Positively set = 3<br>Unknown = 4
**Stay in the NICU: general data, breastfeeding/pumping behavior**	
Open introduction: Your baby/ babies were born prematurely, so I have a few questions about that.	
Were you in the NICU with your baby after he/she was born, and if so, for how long?	Create 3 variables if possible:<br>Stay in NICU (yes=1/no=2)<br>Stay in NICU (yes=1/no=2)<br>Total duration of NICU stay<br><br>Code exact number of days
How long in total were you in the hospital with your baby?	See above

When did you first see your baby after birth?	immediately after birth = 1<br>the same day = 2<br>the following day = 3<br>later = 4
While your baby was in the NICU, were you allowed to see your baby at any time, were your visitation rights restricted?<br><br>If so, what kind? How did you experience these restrictions (e.g., were they burdensome or relieving)?	Yes = 1<br>No, there were fixed times (without inclusion of corona measures) = 2<br>No, there were corona-related restrictions = 3<br>______________________<br>Likert scale 5 levels (not burdened at all = 1, not burdened = 2, neither burdened nor relieved = 3, burdened = 4, very burdened = 5)
Did your baby receive woman's milk (i.e., breast milk or donor milk) from birth?	yes = 1<br>no = 2<br>I do not know = 3
If yes: Did you provide your baby with breast milk yourself, or did your baby receive donor milk?	Own milk = 1<br>Donor milk = 2<br><br>Neither of both = 3
Did you obtain breast milk after birth? If yes: how (express, pump)?<br><br><br><br>Approximately how often and when did you switch to other methods of obtaining breast milk, if applicable?	no = 1<br>yes, by expressing = 2<br>yes, by pumping = 3<br><br>yes, byexpressing and pumping = 5<br>yes, by breastfeeding = 4<br>____<br>Code the number of hours. For 2-3, 3-4, etc., code 2.5; 3.5, etc., respectively.<br><br>No fixed intervals = -77<br>____<br>Change after one day = 1<br>Change after 2 days = 2<br>Change after 3 days = 3<br>Changeover after approx. one week = 4<br>Change only after hospital at home = 5<br>Change when the baby was no longer being probed = 6<br>Change after more than one week = 7
How did you feel during your first attempts at breast milk collection?	proud = 1<br>secure = 2<br>neutral = 3<br>insecure = 4<br>stressed = 5<br>instrumentalised = 6<br>Excited = 7
Have you been educated about the special importance of colostrum for preterm infants?	no = 1<br>yes = 2<br>I do not know = 3
If yes: by whom?	by the nursing staff = 1<br>by the physicians = 2<br>by lactation consultants = 3

	by midwife / midwifery student = 4<br>self informed = 5
Did you pump in the NICU?	no = 1<br>yes = 2
If yes: How soon after birth did you first pump? If no, why did you not pump?	immediately after birth = 1<br>within the first hours after birth = 2<br>within the first day after birth = 3<br>later than 24 hours after birth = 4<br><br>Note: There was no information on No, so no categories were defined here
If yes: Approximately how often did you pump during the first three days? Were there fixed time intervals?	Code the number of hours. For 2-3, 3-4, etc., code 2.5; 3.5, etc., respectively.<br>No fixed time intervals = -77<br><br>Create an additional variable Night breaks to be coded as follows:<br>Night breaks inserted yes= 1<br>Night breaks inserted no = 2
If yes: Did you pump both breasts at the same time or one after the other?	simultaneously = 1<br>one after the other = 2<br>First one after the other, then simultaneously = 3
Were you reminded to pump by physicians, nurses, or other hospital staff?	yes = 1<br>no = 2<br>I do not know more = 3
Were you allowed to see your baby while you pumped?	yes = 1<br>no, generally not = 2<br>no, not possible due to corona = 3<br>don't know = 4
If your baby did not get breast milk from birth, did he or she get your breast milk or donor milk at a later time?	yes = 1<br>no = 2
If yes, from when?	After approx. one day = 1<br>After several days, within the first week = 2<br>Within the first two weeks = 3<br>Only after discharge from the intensive care unit = 3<br>Only after the tube has been removed = 4<br>Only at home = 5<br>Not at all = 6
If donor milk: when were you first able to feed donor milk?	Not applicable, therefore no categories
If breast milk (also after donor milk): When were you first able to feed your own breast milk?<br><br>How was this done (e.g., by tube, by breastfeeding, or by pumping and feeding)?<br><br>Would you have been able to do this sooner?	Within the first day after birth = 1<br>The following day after birth = 2 Within the first week after birth = 3<br>Within the first 2 weeks after birth = 4<br>Later = 5<br>Not at all = 6<br><br>Tube = 1<br>Pumping and feeding = 2<br>Breastfeeding = 3<br><br>yes = 1

	no = 2
Did the hospital give you the opportunity to go to your baby at night and feed him or her your breast milk or donor milk?<br><br>If not, would you have liked to have had such an opportunity?	yes = 1<br>no, but that was also okay = 2<br>no, but I wished that = 3<br><br>yes = 1<br>no = 2
Did you feel you were allowed to make your own decisions regarding breastmilk supply/donor milk supply for your baby and also to say you did not want things you were advised to do (e.g., feed donor milk, use nursing caps, etc.)?	Likert 5 levels<br>1 = not at all self-determined<br>3 = neither<br>5 = absolutely self-determined
Do you feel or have you felt pressured or pushed to breastfeed and pump against your wishes?	Likert 5 levels (does not apply at all = 1, does not apply = 2, neither = 3, applies = 4, applies completely = 5)
If so, by whom?	Physician = 1<br>Partner = 2<br>Nursing staff = 3<br>Midwife / midwifery student = 4<br>Lactation consultant = 5<br>Other person = 6
It can happen that some children in the NICU are not yet in good health at the beginning. Was this the case with your child?<br><br>If so, what were the problems	No complications = 1<br>One complication = 2<br>Multiple complications = 3<br><br>Type:<br>Infection = 1<br>Breathing problems beyond normal level = 2<br>Cerebral hemorrhage = 3<br>Inguinal hernia = 4<br>Digestive problems beyond normal level = 5<br>Intestinal inflammation = 6<br>Other = 7
**External breastfeeding and pumping support:**	
Open introductory question: did anyone help you breastfeed or pump?	yes = 1<br>no = 2
Has your partner assisted you with breastfeeding, expressing, or pumping?	yes = 1<br>partly = 2<br>no = 3
Have you been counseled about the importance of breast milk for preterm infant health? This includes counseling before birth, but also after	yes, before birth = 1<br>yes, after birth = 2<br>no = 3
Were you assisted in breastfeeding or obtaining breast milk in the hospital, for example, by explanations about breast pumps, latching on, etc.?	yes = 1<br>no = 2
If yes: By whom?	by the midwife / student midwife = 1

	by the nursing staff = 2<br>by the doctors = 3<br>by the breastfeeding and lactation consultant = 4<br>Through other mothers = 5
Were you satisfied with breastfeeding and pumping support in the hospital	Likert 5 levels<br>(not at all satisfied = 1, not satisfied = 2, neither nor = 3, satisfied = 4, very satisfied = 5)
What tips or what support for breastfeeding and pumping from the physicians helped you the most?	Instructions for pumping out = 1<br>Instructions for putting on = 2<br>Information about MM supply = 3<br>Encouragement = 4<br>Appreciation = 5
What tips or what support for breastfeeding and pumping from nurses (in neonatology or gynecology wards) and midwives helped you best?	Instructions for pumping out = 1<br>Instructions for putting on = 2<br>Information about MM supply = 3<br>Encouragement = 4<br>Appreciation = 5<br>Other=6
Is there anything you would have liked in terms of advice or support to promote breastfeeding and pumping or breast milk production?	More appreciation = 1<br>More sensitivity / more empathy = 2<br>more information about MM care = 3<br>More direct care and guidance in the process = 4<br>More / longer availability of trained staff = 5<br>Consistent information = 6<br><br>Better availability of necessary infra structure (breast pumps, etc.) = 7
Did you feel comfortable with the physicians' guidance of the breastfeeding process/ pumping?	Likert 5 levels (I felt very uncomfortable = 1, I felt uncomfortable = 2, I felt neither comfortable nor uncomfortable = 3, I felt comfortable = 4, I felt very comfortable = 5)<br><br>No accompaniment by physicians = -99
Did you feel comfortable with the nurses' assistance with the breastfeeding process / pumping?	Likert 5 levels (I felt very uncomfortable = 1, I felt uncomfortable = 2, I felt neither comfortable nor uncomfortable = 3, I felt comfortable = 4, I felt very comfortable = 5)<br><br>No accompaniment by caregivers = -99
Do you have or have you had negative feelings about breastfeeding or pumping? For example, some mothers feel shame, dejection, anger, guilt, or feel like they are failing. Have you experienced these feelings as well?	no = 1<br>yes, one negative emotion mentioned = 2<br>yes, several negative emotions mentioned = 3
If so, what have you done to change this and what has helped you?	Psychological support = 1<br>Support from partner = 2<br>Support from extended family = 3<br>Support from friends = 4<br>Non-psychological support from hospital = 5

	Was able to self-regulate = 6<br>No support, but also no improvement = 7
Did you feel that you or your body was being instrumentalized in terms of breastfeeding and obtaining breast milk? If so, did you feel this was negative or perhaps even necessary in the situation?	no = 1<br>yes, but negative = 2<br>yes, but necessary = 3
Did you feel that you or your body were being reduced to breast milk production?	Likert 5 levels<br>(not at all = 1, rather not = 2, neither = 3, rather yes = 4, yes, very = 5)
Did you have any breastfeeding counseling after your hospital stay?	no = 1<br>yes = 2
If so, by whom?	By the follow-up midwife = 1<br>Through the lactation consultation of the KH = 2<br>By a lactation consultant independent of the KH = 3<br>Through another person = 4
How confident did you feel about breastfeeding/pumping afterward?	Likert 5 levels (not at all sure = 1, not sure = 2, neither sure nor unsure = 3, sure = 4, very sure = 5).
What breastfeeding and pumping tips would you pass on to other mothers?	Do not let others determine = 1 take your time = 2<br>try again and again = 3<br>try home remedies = 4<br>do not lose courage = 5<br>inform yourself = 6
What tips would you have for medical and nursing staff to better assist mothers with breastfeeding / pumping?	do not create pressure = 1<br>sensitive approach to the subject = 2<br>no comparisons = 3<br>more factual information = 4<br>uniform instructions = 5<br>more appreciation = 6<br><br>invest more time / better availability = 7
Did you feel valued in your breastfeeding and pumping efforts?	no = 1<br>yes = 2
If yes: By whom?	By the nursing staff = 1<br><br>By the midwife/ midwifery student = 2<br>Through the physicians = 3<br>Through my family = 4<br>Through my friends = 5<br>Through the lactation consultant = 6<br><br>Mention of more than one person = 7
If yes: Was this appreciation important to you? If applicable, especially in the early days? Why (e.g., motivation)?	Likert 5 levels (not at all important = 1, not important = 2, neither important nor unimportant = 3, important = 4, very important = 5)<br><br>Motivation = 1<br>Well-being = 2<br>Eliminate insecurities = 3<br>Pride = 4

Did you feel confused by conflicting information about breastfeeding and pumping from different sources?	no = 1<br>yes = 2
If yes: Do you have examples of conflicting information?	There were contradictory statements about the pump-down/breastfeeding frequencies = 1<br>There were conflicting statements about pumping/breastfeeding times = 2<br>There were conflicting statements about when to start breastfeeding = 3<br>Other = 4
How do you feel while breastfeeding or pumping?	Divide into positive emotions (13a) and negative emotions (13b)<br><br>13a: pos<br>Likert 7 levels<br>not present at all=1<br>not present = 2<br>rather not present = 3<br>Neither = 4<br>rather present = 5<br>present = 6<br>very strongly present = 7<br><br>13b: neg<br>Likert 7 levels<br>not present at all=1<br>not present = 2<br>rather not present = 3<br>neither = 4<br>rather present = 5<br>present = 6<br>very strongly present = 7
What do you think would have to be done to convince mothers to breastfeed especially premature babies or to pump breast milk for them?	take away their stress/pressure = 1<br>educate about benefits of breast milk = 2<br>provide more offers of help = 3<br>offer more trained counselors = 4<br>offer psychological help = 5
What do you think would have to be done to convince mothers to accept donor milk for their babies?	Education about advantages = 1 Provide education on hygiene, etc. of donor milk = 2<br>Offer more trained counseling centers = 3
What form of support or advice from physicians and nurses do you think is indispensable / most important / for breastfeeding or providing breast milk for premature babies?	Motivate = 1<br>Taking time = 2<br>Psychological counseling = 3<br>Medical counseling = 4<br>appreciation = 5<br>Giving uniform information on breastfeeding behavior = 6
How easy did you find it to continue pumping/breastfeeding after discharge?	Likert 5 levels (1 = not at all easy, 3 = neither, 5 = very easy)
Did you face any obstacles in doing so?	No obstacles = 1<br>No more (weight/quantity) Control = 2<br>too little milk = 3<br>Difficulties with latching on = 4

	Difficulties with availability of breast pump = 5<br>Time problems due to effort with the hygiene of the pump sets etc. = 6
What support did you find or would you find helpful to continue breastfeeding / pumping at home after your stay in the hospital?	Support and counseling services provided by the KH = 1<br>Breastfeeding and lactation counseling for the home = 2<br>Talks with the aftercare midwife = 3<br>Possibility of telephone consultation = 4<br>Help to make the necessary infrastructure available (breast pump, pump sets, etc.) = 5<br>More information in advance = 7<br><br>If more than one factor is mentioned, code the one to which the woman attaches the greatest importance.
What information do you think is important when it comes to breastfeeding/breastmilk supply in general and especially breastfeeding/breastmilk supply for premature babies?	Information on medical factors (e.g. milk production, disease prevention, etc.) = 1<br>Information about it being the best possible diet = 2<br>Information about special bonding / closeness to the child = 3<br>Information about support options in case of problems = 5
How did you get information about breast milk and breastfeeding, what were your main sources of information?	Hospital staff = 1<br>Friends/acquaintances = 2<br>Family = 3<br>Professional journals = 4<br>Websites = 5<br>Books = 6<br>Other mothers in the hospital = 7<br>Midwife = 8<br>Gynecologist (external, not hospital) = 9<br>Birth preparation course = 10
Did you network and share with other breastfeeding mothers?	no = 1<br>yes = 2
If yes: online or in real life?	Online (Whatsapp and internet in general) = 1<br>Real in the hospital = 1<br>Real in the waiting room at the physician = 3<br>Real at private meeting = 4Real at private meeting = 4
Did these exchanges help you?	Likert 5 levels (not helped at all = 1, helped a little = 2, neither = 3, helped a little = 4, helped a lot = 5).
If yes: what exactly and in what ways?	Emotional support = 1<br>Information acquisition (e.g. problem solving) = 2<br>Motivational support = 3<br>Gain of security = 4
	no = 1<br>yes, when the amount of milk was sufficient = 2<br>yes, the first time I breastfed = 3<br>yes, when I was allowed to take over tasks = 4

	yes, when my child made developmental progress = 5
**<u>Psychological stress factors</u>**	
Did you feel psychologically burdened by the premature birth and everything that went along with it?	Likert 5 levels (not at all burdened = 1, little burdened = 2, neither = 3, rather burdened = 4, very burdened = 5).
If yes: By what in particular?	Self-blame because of / processing problems of premature birth in general = 1<br>pressure around breast milk = 2 postoperative delirium = 3<br>separation from the child = 4<br>Loneliness due to hospitalization / corona restrictions = 5<br>Medical problems of the child = 6
Do you currently feel particularly psychologically stressed?	Likert 5 levels (not at all burdened = 1, little burdened = 2, neither = 3, rather burdened = 4, very burdened = 5)
If yes to one of the two questions: Did your psychological stress situation prevent you from providing your baby with breast milk?	no = 1<br>yes = 2
If yes: Through whom did you get help or are you getting help?	Lactation consultant = 1<br>Psychologist = 2<br>Nursing staff = 3<br>Physician = 4<br>Partner = 5<br>Extended family = 6<br>Friends = 7<br>Others = 8
If you would like to share: If applicable, have you received a diagnosis of mental illness?	no = 1<br>yes = 2
If yes: when and which one?	Postpartum depression = 1<br>PTSD = 2<br>depressive episode = 3<br>other = 4
Has breastfeeding or pumping helped you cope with your psychological distress situation?	Likert 5 levels (not helped at all = 1, helped a little = 2, neither = 3, helped a little = 4, helped a lot = 5)
**<u>Gender role orientation</u>**	
Do you see breastfeeding as a natural duty of a mother?	Likert 5 levels (not at all true = 1, not very true = 2, neither nor = 3, somewhat true = 4, very true = 5).
Does your partner see breastfeeding as a mother's duty?	Likert 5 levels (not at all true = 1, not very true = 2, neither = 3, somewhat true = 4, very true = 5)
Does your partner help with household chores?	no = 1<br>helps a little = 2<br>yes (approximately equal distribution) = 3
Does your partner help with the care of your baby/children in everyday life?	no = 1<br>helps a little = 2<br>yes (approximately equal distribution) = 3
**<u>Past breastfeeding and pumping behavior</u>**	
Do you have other children?	no = 1<br>yes = 2

If so, how old are they?	Code age in years
Were your other children also born prematurely?	no = 1<br>yes = 2<br><br>Note: If only one of the other children was born prematurely and the others were not, code yes anyway.
Did you breastfeed your other children or pump breast milk for them?	no = 1<br>yes, but not all = 2<br>yes, all = 3
If you had leftover breast milk yourself, would you have provided it as a breast milk donation for other babies, or might you have actually donated breast milk?	no = 1<br>yes would have donated = 2<br>yes, has donated = 3<br>Do not know = 4
**<u>Breastfeeding apps</u>**	
Open introductory question: are you familiar with breastfeeding support apps?	no = 1<br>yes = 2
If known: Do you use a breastfeeding support app yourself?	no = 1<br>yes = 2
If yes: Which one?	Baby Connect = 1<br>MyMedela = 2<br>Ovia = 3<br>Baby Daybook = 4<br>Baby Tracker = 5<br>Babyplus = 6
If no: Would you use a breastfeeding support app?	no = 1<br>yes = 2<br>I do not know = 3
How likely is it (in percent) that you would use a breastfeeding support app?	Code the exact percentage. If the woman has specified a from-to number, select the middle (e.g. 80 - 90 % = 85).
What features would you want in such an app?	Individual timer (reminder) for pumping / breastfeeding= 1<br>Note window = 2<br>Documentation weight of the child = 3<br>Documentation amount of milk = 4<br>Documentation breast was pumped/with which side breastfed? = 5<br>Option of entering milestones = 6<br>Photo album = 7<br>Direct link to counseling center = 8
How often or at what intervals should such an app remind them to breastfeed?	Individually adjustable = 1<br>Every 2 hours = 2<br>Every 2 - 3 hours = 3<br>Every 3 hours = 4<br>Every 3 - 4 hours = 5<br>Every 4 hours = 6<br>Greater than 4 hours = 7

What information about breastfeeding and pumping should be included in such an app in any case?	general information on MM = 1<br>general information on pumping = 2<br>general information on breastfeeding = 3<br>information on childhood illnesses = 4<br>information on parental allowance etc. = 5
**Sociodemographic questions**	
How old are you?	Code exact age
Were you born in Germany?	yes = 1<br>no = 2
If no: In which country were you born?	Code as string variable and enter country
If no: How long have you lived in Germany?	Does not need to be coded, as inapplicable
Are you in a relationship? If yes: With the father of the child or with another person / Do you have a husband/wife, boyfriend or girlfriend? If husband/boyfriend: Is he the father of your child?	No = 1<br>Yes, with the father of the child = 2<br>Yes, with another person = 3
If married/partnered, how many years have you been married/partnered?	Create two variables here and code duration of marriage and duration of relationship total, if the person is not married code -99 for marriage.
If no: Are you a single parent or which parenting model between partners do you use / Do you take care of your child alone or together with your partner?	Sole custody = 1<br>Alternating model = 2<br>Separate but basically joint parenting = 3<br>Shared custody, but sporadic contact with the father = 4
Do you belong to a religious group?	no = 1<br>yes = 2
If yes, which one?	catholic = 1<br>Protestant = 2<br>muslim = 3<br>jewish = 4<br>other = 5
What level of education do you have?	None = 1<br>Secondary school = 2<br>Realschule = 3<br>Vocational baccalaureate = 4<br>high school diploma = 5<br>Completed studies = 6
Are you employed / Do you have a job?	no = 1<br>yes = 2
If yes: What is your current employment status? (e.g. full-time / part-time / mini-job)	Full time = 1<br>Part time = 2<br>Minijob = 3
What was your last occupation / do you currently practice?	Create as string variable and enter profession
If employed: Have you already returned to your job?	no = 1<br>yes = 2
If yes: How old was your child when you started working again?	Create as string variable and enter profession
If back at work: Why did you start working again? (e.g., financial reasons, enjoyment of work, securing position, etc.?)	Financial reasons = 1<br>Fun at work = 2<br>Secure position = 3<br>Self-employment = 4

	Help for the employer = 5<br>Variety = 6
If still breastfeeding/pumping and working: Does your employer support breastfeeding/pumping or provide you with the necessary breaks?	no = 1<br>yes = 2
If still breastfeeding/pumping and working: How do you handle breast milk supply while you are at work? / How do you handle feeding breast milk while you are at work?	Pump down and bottle by third party = 1<br>Pumping and bottle on site = 2 Breastfeeding on site = 3<br>Powder milk feeding by third party = 4<br>Powder milk feeding on site = 5
At what point in your pregnancy did your baby come into the world? Please give your answer in the following format, e.g. 37 + 5. If you do not know the exact gestational age, give only the completed week of pregnancy: 31 here means 31 completed weeks, so birth at 32 weeks.	Coding at 37 + 5 as 37.5, at 22 + 1 as 22.1, etc.
When did you know that your baby would be born prematurely?	A few weeks before = 1<br>A few days before = 2<br>one day before = 3<br>some hours before = 4<br>Not at all = 5
How old is your baby now? If known, feel free to list both the real age and the corrected age.	Create two variables, one for real age and one for corrected age, each entered in months. For half months, select decimal place .5
